# Predators and dispersers: Context-dependent outcomes of the interactions between rodents and a megafaunal fruit plant

**DOI:** 10.1038/s41598-020-62704-6

**Published:** 2020-04-08

**Authors:** Andrea P. Loayza, Claudia A. Luna, María Calviño-Cancela

**Affiliations:** 1 Instituto de Investigación Multidisciplinario en Ciencia y Tecnología, Universidad de La Serena, La Serena, Chile; 2 Instituto de Ecología y Biodiversidad (IEB), Las Palmeras, 3425 Ñuñoa Chile; 3 Universidad de La Serena, Departamento de Biología, 1305 Raúl Bitrán La Serena, Chile; 4 Universidad de Vigo, Departamento de Ecología y Biología Animal, Facultad de Biología, Vigo, España

**Keywords:** Behavioural ecology, Ecological modelling, Conservation biology, Population dynamics

## Abstract

Many plant species bear fruits that suggest adaptation to seed dispersal by extinct megafauna. Present-day seed dispersal of these megafaunal plants is carried out by rodents, which can act as predators or dispersers; whether this interaction is primarily positive or negative can depend on the context. Here, we parameterized a stochastic model using data from the field and experimental arenas to estimate the effect of rodents on the recruitment of *Myrcianthes coquimbensis* -an Atacama Desert shrub with megafaunal fruits- and examine whether environmental conditions can alter the sign and strength of these rodent-plant interactions. We show that the outcome of these interactions is context-dependent: in wet conditions seed removal by rodents negatively impacts the recruitment probability of *M. coquimbensis*; in contrast, in dry conditions, the interaction with rodents increases recruitment success. In all cases, the strength of the effect of rodents on the recruitment success was determined mainly by their role as dispersers, which could be positive or negative. This study demonstrates that by caching seeds, rodents can be effective dispersers of a megafaunal fruit plant, but that the sign and magnitude of their effect on recruitment changes as a function of the environmental context in which the interaction occurs.

## Introduction

Many New World plant species bear fruits with trait combinations that suggest adaptation for seed dispersal by the megafauna that went extinct during the Pleistocene-Holocene transition^1–5^. Plants with this megafaunal dispersal syndrome bear fleshy fruits too large to be swallowed and dispersed by extant frugivores; hence they are believed to represent seed dispersal anachronisms^2^. In present times, seed dispersal of Neotropical anachronic plants is carried out by surrogate dispersers; smaller-bodied animals, such as scatter-hoarding rodents^4,6^, which can benefit not only from the seed, but also from the fleshy pulp of some anachronic fruits, which provides a sugar- and water-rich reward not produced by typical rodent-dispersed, nutlike fruits^7,8^. Although it is hypothesized that these smaller, surrogate dispersers are unlikely to compensate for the loss of the dispersal services provided by the extinct megafauna^9^, few empirical studies have examined their effectiveness as dispersers of anachronic plants^6,10,11^.

Scatter-hoarding rodents store intact fruits and/or seeds in many separate, shallow caches in the soil^12–15^, and because they generally store more food than they require^16^, they forget or neglect some of the caches^6,15^. Moreover, during the caching and re-caching process, seeds can be moved far from the parent plant^6^ and to habitats with high probabilities of seedling establishment^10,17^. Therefore, seed dispersal by scatter-hoarding rodents can positively contribute to the recruitment of megafaunal fruit plants^4,6,17^. The interaction between these plants and scatter-hoarding rodents, however, also comes with a cost because rodents are seed predators^18^ that eventually recover many of the seeds they dispersed, and partially or completely consume them^14,17,19^. Consequently, the relationship between megafaunal fruit plants and scatter-hoarding rodents is partly antagonistic, due to the role of rodents as seed predators, and partly mutualistic, due to their role as seed dispersers. Whether this interaction is primarily mutualistic or antagonistic may largely depend on the context.

It is generally recognized that the ecological context can determine the outcome (i.e., strength and sign) of species interactions^20–23^. Thus, spatial and temporal variability in the biotic or abiotic environment may not only influence the effectiveness of scatter-hoarding rodents as dispersers by modifying quantitative and/or qualitative aspects of their dispersal services^24^, but also switch the sign of the rodent-plant interaction (from positive to negative or vice versa). Changes in the magnitude and sign of the interaction can be mediated by seed size and by the suitability of the caching habitats, which in turn change in relation to the environmental conditions^25,26^. For example, preferred caching habitats may be more favorable for recruitment in some years (e.g., rainy years) and less in others^26,27^, and the suitability of these sites may change depending on seed size. Therefore, the interplay between seed size and the effect of environmental variability on habitat suitability can determine seed fate and the quality of the dispersal services provided by rodents. Taking this context-dependence into account is essential to assess the net contribution of surrogate dispersers to the long-term population persistence of anachronic plants.

*Myrcianthes coquimbensis* is an endangered, narrow endemic shrub of the southern edge of the Atacama Desert^28^. This region is characterized by having a strong north–south gradient in aridity and high inter-annual variability in rainfall (CV 82.9%)^29^, which results in high spatio-temporal variability in its biotic and abiotic environment. *M. coquimbensis* bears large (2.5 × 3.5 cm), fleshy fruits, typically enclosing a single large, recalcitrant seed. There are no present-day frugivores, nor have there been contemporary losses of large, fruit-eating vertebrates along its area of distribution, which suggests that its fruits evolved in response to interactions with the extinct megafauna of the area (e.g., members of Gomphotheriidae, Mylodontidae, and Macraucheniidae, among others)^30^. The only vertebrates that consume *M. coquimbensis* fruits are three species of scatter-hoarding rodents (differing in body size and behaviour) that feed on the pulp and seed; consequently, they act both as predators and dispersers^17^. Foraging behaviour of these rodents is strongly influenced by *M. coquimbensis* seed size, with larger fruits/seeds having higher probabilities of being harvested and cached^31^. Seed size exhibits large intraspecific variation (0.03–14.6 g) and is positively related to emergence probability. Because seeds are desiccation-sensitive, no seedlings recruit from seeds cached in open interspaces; present-day seedling emergence is restricted to caching sites that are sheltered from the harsh environmental desert conditions, such as in rock cavities and beneath conspecific shrubs^26^. In addition, recruitment in *M. coquimbensis* is promoted by intraspecific facilitation^26^, which overrides the effects of negative density-dependent processes predicted by the Janzen–Connell model^32,33^.

Our primary goal in this study, is to evaluate the effect of each rodent species on the recruitment probability of *M. coquimbensis* and to assess the degree to which seed size preferences and microhabitat of seed arrival interact with environmental conditions (wet vs. dry) to determine the outcome, in both strength and sign, of these plant-rodent interactions. We predict that because of their size and behavioural differences (1) rodents contribute differently to the recruitment of *M. coquimbensis* and (2), independent of the species, the outcome of the rodent-plant interaction will be mediated by the context, with rodents having a more positive effect on recruitment in dry than in wet conditions, because in these conditions, caching seeds in sheltered microhabitats can reduce desiccation and promote seedling recruitment.

## Results

### Context-specific recruitment success of *M. coquimbensis*

**Figure 1:**
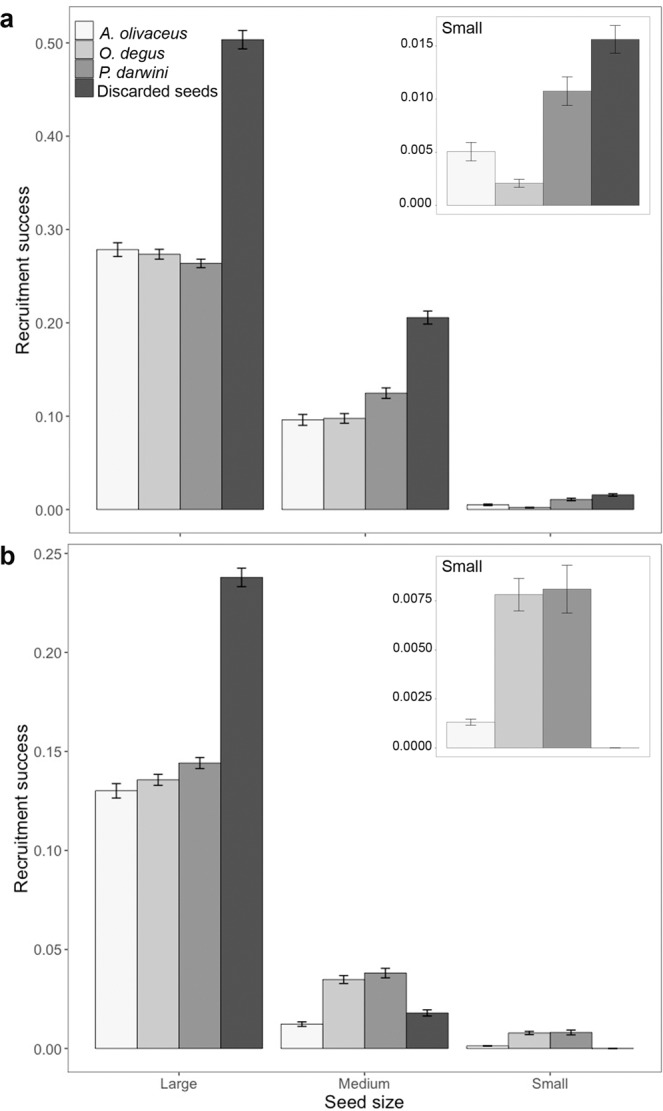
Effect of dispersal or seed consumption by *Abrothrix olivaceus*, *Phyllotis darwini* or *Octodon degus* on the recruitment success of large, medium and small *M. coquimbensis* seeds in wet (**a**) and dry (**b**) conditions. Recruitment success is expressed as the proportion of seeds that successfully emerge as seedlings and is compared to that of discarded seeds.

Environmental conditions, seed size and handling behaviour by rodents were all significant in determining the recruitment success of *M. coquimbensis* (Fig. 1; Table 1). Specifically, the probability of recruitment was 2.44 times higher in wet than in dry conditions (0.159 ± 0.002 vs. 0.065 ± 0.001, respectively), 3.15 times higher for large (0.245 ± 0.003) than for medium seeds (0.078 ± 0.002), and 12.3 times higher for medium than for small seeds (0.006 ± 0.0003). With regard to the effect of handling on recruitment success, we found that seeds discarded beneath the parent plant after being defleshed had the highest (0.163 ± 0.004) probability of recruitment (1.6–1.8 times higher than seeds consumed or dispersed by rodents), followed by seeds selected (consumed or dispersed) by *P. darwini* (0.102 ± 0.002), *O. degus* (0.092 ± 0.002) and *A. olivaceus* (0.090 ± 0.003). In addition, there was a significant interaction between seed size and environmental conditions (Table 1); decreasing seed sizes heightened the negative effect of dry conditions on recruitment success (Fig. 1). Interestingly, the relative effect of seed handling by rodents was also context dependent, varying in relation to environmental conditions and seed size (Fig. 1). For large seeds, those selected by rodents always had lower recruitment success than discarded seeds, in both wet and dry conditions and for all rodent species. In contrast, for medium and small seeds, this differed between wet and dry conditions (Fig. 1): in wet conditions, seeds selected by rodents had lower recruitment success than discarded seeds, whereas in dry conditions the opposite pattern was observed. Among rodent species, *P. darwini* had a slightly more positive effect than the other species on the recruitment success of the medium seeds they selected in wet conditions; this positive effect increased with small seeds. In dry conditions, medium and small seeds selected by *P. darwini* and *O. degus* had similar recruitment success, which was higher than that of seeds selected by *A. olivaceus* and of discarded seeds. The only rodent species that led to lower recruitment of selected compared to discarded seeds of medium size was *A. olivaceus*. For small seeds in dry conditions, all rodent species had a positive effect on recruitment of selected compared to discarded seeds, given that small, discarded seeds had no recruitment (Fig. 1). In summary, the effect of rodents on recruitment success of *M. coquimbensis* became more positive in more unfavourable (i.e., dry) conditions and with decreasing seed size.

**Table 1:** Results of the effect of environmental conditions (Wet/Dry), seed size and seed handling (discarded seeds versus seeds handled by each rodent species) on the recruitment success of *M. coquimbensis* using a Generalized Linear Model with binomial error distributions and logit link function.

Source of variation	d.f.	Deviance (χ^2^)	*P* value
Wet/Dry	1	265.23	<0.001***
Seed size	3	100.57	<0.001***
Seed handling	2	655.43	<0.001***
Wet/Dry: Seed size	2	24.56	<0.001***
Wet/Dry: Seed handling	3	8.20	0.042*
Seed size: Seed handling	6	11.37	0.078 ^ns^
Wet/Dry: Seed size: Seed handling	16	61.5	<0.001***
Residual	11682	855.0	
Total	11705	2652.2	

### Context-specific role of rodents as predators versus dispersers

**Figure 2:**
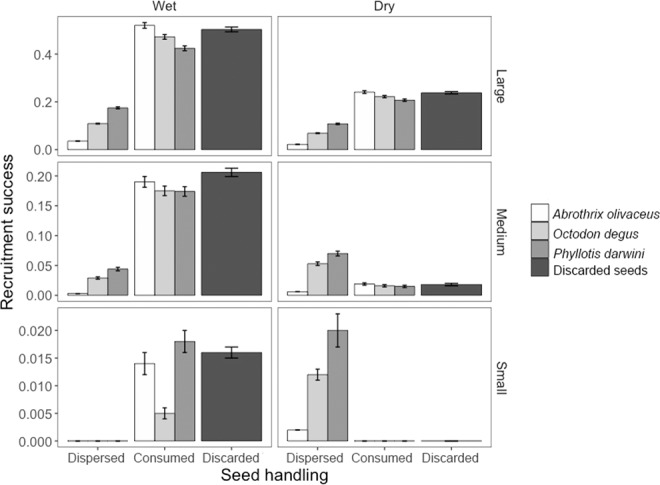
Recruitment success of large, medium and small *M. coquimbensis* seeds consumed *in situ* or dispersed by *Abrothrix olivaceus*, *Phyllotis darwini* and *Octodon degus* in wet and dry conditions. Recruitment success is expressed as the proportion of seeds that successfully emerge as seedlings and is compared to discarded seeds (i.e., intact seeds left under the parent plant).

As shown in Fig. 2, the context-specificity described above (Fig. 1) was primarily a consequence of the recruitment success of dispersed seeds, which differed from that of discarded seeds, and also varied markedly depending on the context. In contrast, recruitment success of seeds consumed *in situ* was similar to that of discarded seeds in all circumstances (except for small seeds consumed by *O. degus* in wet conditions, which had much lower recruitment; Fig. 2). Therefore, in wet conditions, the observed lower recruitment success of seeds selected by rodents compared to discarded seeds (Fig. 1) was mainly a consequence of the lower success of dispersed seeds, particularly small seeds, which did not recruit when dispersed (Fig. 2). Similarly, the positive effect of rodents on recruitment of small and medium seeds in dry conditions, especially of *P. darwini* and *O. degus*, was a consequence of their positive effect as dispersers; they had no negative effect as predators since seeds consumed *in situ* by these two species had a similar recruitment success to discarded seeds (Fig. 2).

**Figure 3:**
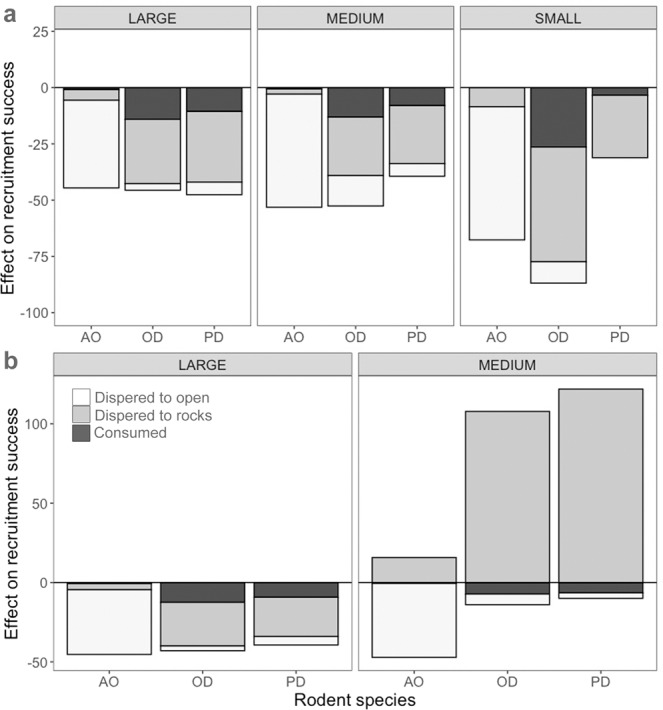
Effect of *Abrothrix olivaceus* (AO), *Phyllotis darwini* (PD) and *Octodon degus* (OD), on the recruitment success of large, medium and small *M*. *coquimbensis* seeds expressed as the percentage increase or decrease compared to the fate of discarded seeds (i.e., intact seeds left under the parent plant) in wet (**a**) and dry (**b**) conditions. Shades of grey represent the relative proportion of the effect resulting from seed consumption (*in situ* or at the caching sites) and from seed dispersal to open areas and rocks. No bars are shown for small seeds in dry conditions because, while there was emergence of handled seeds in these circumstances, there was no emergence of discarded seeds, making the effect of rodents on recruitment infinitely positive, and the percentage of this effect corresponding to predation and dispersal being impossible to estimate.

To summarize the effect of seed handling by each rodent species (i.e., their quality) as a percentage increase or decrease in recruitment success compared to discarded seeds in each circumstance: in wet conditions, the net effect of rodents on recruitment success was negative (Fig. 3), ranging between 39% to 53% for large and medium seeds and reaching a maximum of 87% (with *O. degus*) for small seeds. In dry conditions, the net effect of rodents was again negative for large seeds (about 40%), but shifted towards positive for medium seeds handled by *O. degus* and *P. darwini* (Fig. 3), and for all small seeds handled by any of the rodent species (not represented in Fig. 3, as this effect tended towards infinity due to the lack of recruitment of discarded seeds). In all cases, the strength of the effect of rodents on recruitment success was determined mainly by their role as dispersers, while their effect as predators was relatively minor (reaching a maximum of 30% of the net effect of each rodent; Fig. 3). The effect of rodents was mainly mediated by dispersal to open sites in the case of *A. olivaceus*, and by dispersal to rock cavities in the case of *O. degus* and *P. darwini* (Fig. 3). Both *in situ* consumption and dispersal to open sites (where seedling emergence was nil) had a negative effect on recruitment success in all circumstances. In contrast, the effect of dispersal to rock cavities was negative or positive depending on the context: it was negative in wet conditions for all seed sizes and in dry conditions for large seeds, but positive for medium and small seeds in dry conditions (Fig. 3).

## Discussion

We show the complex relationships of a rodent-mediated seed dispersal system. Our results revealed that, by caching seeds, scatter-hoarding rodents can provide effective dispersal services to a megafaunal fruit plant, but that the sign and magnitude of their effect on plant recruitment changes as a function of the context in which the interaction occurs. To our knowledge, this is the first study to examine how the context can alter the outcome of the interaction between rodents and a megafaunal fruit plant.

As predicted, each rodent species had a different effect on the recruitment probability of *M. coquimbensis* and this effect was markedly context-specific. Rodents contributed more to recruitment in dry, than in wet conditions. The context specificity was mainly mediated by their role as seed dispersers, while their effect as seed predators was small (evidenced by small differences in recruitment success of consumed seeds compared to discarded seeds left intact under the parent plant). This reduced effect of predation is explained because seed consumption in *M. coquimbensis* is seldom lethal; seeds are usually only partially consumed and can lose *ca*. 90% of their mass and still emerge^17^. Therefore, the recruitment success of this endangered plant is determined more by the microhabitat of seed caching than by predation and, because the suitability of microhabitats changes dramatically from wet to dry conditions, recruitment success of *M. coquimbensis* becomes context-dependent. For fruits dispersed to caching sites, rodents consume the pulp and store intact seeds for later; they may later recover and consume the seeds, but again, the usual partial consumption constraints the effect of predation, making the effect of the microhabitat of seed arrival more decisive.

Differences in the quality of dispersal among rodent species are mainly determined by their patterns of habitat use. In general, *P. darwini* and *O. degus* cache seeds in rock cavities, where seeds have high probabilities of establishment. In contrast, *A. olivaceus* caches seeds more frequently in open sites, where seedlings cannot recruit^26^. Predation risk is one of the main determinants of habitat use by rodents and is influenced by rodent body size, since local predators prefer larger rodent prey^34^. The smaller size of *A. olivaceus* (30 g, in contrast with the 60 g of *P. darwini* and 140 g of *O. degus*) favours its use of risky, open sites^35^. Thermoregulatory constraints further limit the use of open sites by diurnal species such as *O. degus* (*P. darwini* is nocturnal) in this arid ecosystem, which tend to avoid open sites even when predators are absent^36^. The harsh conditions in open sites also restrict recruitment to nurse microhabitats, such as shrubs or rock cavities, where soil and air temperatures are lower and water availability is higher than in open interspaces^26^. Under conspecific shrubs, the large amounts of leaf litter likely ameliorate soil conditions, increase seed longevity and reduce early seedling mortality from desiccation^26,37^. Conspecific shrubs, however, may also exert intensive competition for resources with recruits. This would explain the context-dependence observed in the effect of dispersal to rocks (see Fig. 3), since increased competition for soil water under dry conditions can reduce the nurse effect of conspecific shrubs, especially for smaller seeds, because small seed size positively correlates with the rate of dehydration and ultimately seed lifespan^38^. Thus, while in wet conditions recruitment is higher under *M. coquimbensis* plants, in dry conditions only large seeds recruit better under conspecifics, while smaller seeds are more likely to recruit in rock cavities than under conspecific plants. This marks the shift observed in the effect of rodents, from negative to positive under harsher conditions, because in these circumstances the better conditions for recruitment in rock crevices compensate for the effects of seed consumption and dispersal to open sites (which are always negative). The effect of competition for resources under conspecifics can also reduce survival of new recruits in further ontogeny stages, resulting in reduced suitability of this microhabitat in the long term compared to rock cavities. This would explain the observed higher abundance of seedlings in rocks compared to under conspecifics^17^. Moreover, environmental (i.e., dry/wet) conditions and seed size are not independent, but interact; with seed sizes tending to decrease with decreasing water availability (P. García-Guzmán, unpublished data), probably because seed mass of recalcitrant seeds increases right up to the time of seed fall^39^. This reinforces the positive effect of rodents with increasing aridity.

This study makes two important assumptions. First that rodent foraging behaviour does not change in wet and dry conditions; environmental conditions, however, could alter seed caching and consumption behaviour through resource availability^16^. When food is scarce (i.e., in dry conditions), animals are more likely to consume, rather than cache seeds^40,41^. Nonetheless, there is also evidence that as resource availability decreases, hoarders invest more heavily in cache protection and cache seeds further away^42^. Thus, dry conditions may exacerbate the role of rodents as predators, but seeds that are cached further away, may be less likely to be pilfered and have higher survival probabilities. Finally, it is also possible that because rodent populations in dry conditions are strongly reduced^43^, *per capita* seed availability does not change in relation to wet conditions, and consequently does not lead to changes in behaviour. The second assumption is that rodent behaviour in captivity can be used as a proxy for behaviour in the wild. Although our experimental arenas do not wholly reproduce the environment where these rodents inhabit, an experimental setup is necessary when testing hypotheses that are difficult to examine under natural conditions^44^. Here, we demonstrate that the sign and magnitude of the interaction between rodents and a megafaunal plant can change depending on the context; further field studies can strengthen our results and examine the consequences of a changing environment on the foraging behaviour in the wild. Nonetheless, our results put forward a direct implication: namely that the ecological context can determine the outcome of species interactions.

Context-dependent variation in rodent-plant interaction outcomes coupled with variation in environmental conditions both in time and space are expected to have important consequences on plant population dynamics. The area of distribution of *M. coquimbensis* is especially interesting in this regard because, on the one hand, it is influenced by El Niño, which translates into high inter-annual variation in rainfall, and on the other, it is located within a spatial gradient (North-South) in aridity. Our results suggest that during higher-than-average precipitation years associated with El Niño events^45,46^, when conditions are in general more favourable for *M. coquimbensis* seedling recruitment, seed removal by rodents negatively impacts the probability of recruitment of individual seeds; in contrast, during dry years, when overall recruitment is very limited, the interaction with rodents increases recruitment success of the seeds they selected. Consequently, it is when environmental conditions become more severe for recruitment, when the role of rodents becomes important for population growth. Changes in frequency, duration and magnitude of El Niño events are expected as a consequence of on going global climate change^42^. During the 20th century, rainfall along the study area averaged 170 mm/year; currently, this average has decreased by half, ranging from 21 to 122 mm annually (81 ± 28 mm for the past 15 years, mean ± SD, CEAZAMET). If this drying trend continues, seedling recruitment will be severely reduced; in this harsher scenario, rodents, particularly *P. darwini* and *O. degus*, will become increasingly more important for the regeneration of *M. coquimbensis*.

The restricted distributions of many plants with an anachronic seed dispersal syndrome^47,48^, suggests to some extent dispersal failure^48^. Although, the fruits of some of these species may be able to establish beneath the parent plant^49^, without seed dispersers megafaunal fruit plants will ultimately face declines in their distribution^50^, particularly in areas facing high rates of habitat loss, such as Mediterranean Chile^51^. In this light, seed dispersal by surrogate dispersers, such as rodents, may be key towards increasing their probability of persistence in the Anthropocene. However, the role of surrogate dispersers on the regeneration of megafaunal fruit plants is just beginning to be evaluated^6,52–56^ and empirical evidence is largely biased towards tropical areas, while much less is known for arid ecosystems, which face an exacerbated risk regional warming, water deficit and ultimately land degradation in the near future^57^. Our understanding of the roles that rodents may play on population dynamics of anachronic plants in these, and other environments, will rely on understanding the multiple roles (beyond seed dispersal) they have with their interacting species, and also how these roles are shaped by the environmental context.

## Methods

### Study system

*Myrcianthes coquimbensis* (Barneoud) Ladrum & Grifo (Myrtaceae) is an evergreen shrub restricted in geographic distribution to a coastal strip 83 km long by approximately 1 km wide in the southern limit of the Atacama Desert^28^. This species is a tropical relict and the only member of the genus *Myrcianthes* in Chile; its closest relatives are found in tropical forests of Bolivia, Brazil and Argentina^58,59^. *M. coquimbensis* is distributed along a gradient of decreasing rainfall, with its northernmost populations receiving around 45 mm annually, the centre populations around 70 mm and the southern populations approximately 90 mm (averages from 2011–2014)^60^. In addition to this geographic variation, precipitation also varies considerably among years, with wet years associated to El Niño-Southern Oscillation events^45^. Overall, however, this species grows in an extremely arid environment, where dry conditions prevail.

This shrub produces large (2.5 × 3.5 cm), red, fleshy fruits bearing a sugar- and water-rich pulp. Each fruit typically bears a single seed ranging in mass between 0.05 and 14.58 g; however, a single fruit can have up to four seeds^31^. Fruits are consumed solely by three species of seed caching rodents: *Abrothrix olivaceus* [30 g], *Phyllotis darwini* [60 g] and *Octodon degus* [140 g]^17^. Since rodent-dispersed plants typically bear large, smooth, ovoid nut-like fruits often exhibiting earth tones^18^, and closely related Myrtaceae in tropical areas are not dispersed by rodents but by large frugivores (e.g., primates)^61^, it is unlikely that rodents were the original dispersers of this species. Instead, they likely represent surrogate dispersers^6^ of the extinct Pleistocene megafauna that inhabited the area^30^.

When encountering a fruit beneath the mother plant, rodents can ignore the fruit completely or handle it. Seeds from ignored fruits do not recruit because pulp removal is necessary for seedling emergence (Loayza, *unpublished data*), hence these fruits were not considered in this study. Alternatively, when rodents handle a fruit, they may: 1) consume the pulp and discard the intact seed at the point of encounter (discarded seeds, hereafter), 2) consume the entire fruit and seed, or 3) remove whole fruits or defleshed seeds and cache them away from parent plants (i.e., disperse the seeds). Dispersed seeds are cached predominantly in crevices within rock outcrops and less frequently in open interspaces, while discarded seeds remain beneath mother plants. When rodents recover fruits from the caching sites, they first consume the pulp and can either re-cache the seed, or consume it (totally or partially). In *M. coquimbensis*, the loss of cotyledonary reserves by partial seed consumption is not lethal, and large seeds can lose *ca*. 90% of their mass and still emerge^17^.

### Model parameterization

To assess the effect of rodents on *M. coquimbensis* recruitment, we used stochastic simulations to compare the recruitment success of discarded seeds left beneath the parent plant and that of seeds predated or dispersed by each rodent species. We defined recruitment success as the percentage of seeds that successfully emerged as seedlings. The model (detailed in the next section) was parameterized using data collected in experimental arenas and in the field.

#### Rodent behaviour

We assessed rodent foraging behaviour by conducting cafeteria experiments, which are detailed in Luna *et al*.^31^. Briefly, 10 rodents of each species were captured in the field, taken to Universidad de la Serena and individually placed in 2.5 × 2.5 × 0.5 m experimental arenas. Arenas were covered with a10 cm layer of soil, and had a group of freshly cut *M. coquimbensis* branches in the middle to simulate a shrub, and two plastic hamster houses (located on opposite sides) to simulate sheltered sites. Once rodents had acclimated to the arena, we placed 30 individually marked fruits (10 large [>6 g], 10 medium [3.1–6 g] and 10 small [0.1–3 g]) underneath each *M. coquimbensis* shrub (their only food source) and the trial began. Each trial lasted four days: for *A. olivaceus* and *O. degus* (diurnal species), trials begun in the morning (9:00 a.m.), whereas for *P. darwini* (the nocturnal species) trials started in the evening. Rodents went through a trial individually and only once. At the end of the trial, we recorded the fate of each fruit, specifically: (1) whether it was ignored or handled; (2) if it was handled, whether seeds were discarded beneath the parent plant or selected for caching or consumption; (3) the microhabitat where fruits/seeds were cached; (4) whether seeds were intact, partially or completely consumed and; (6) the percentage of seed mass loss of all partially consumed seeds. With these data we calculated for each size class the probability: (1) that a fruit was ignored, consumed at the encounter site or cached; (2) that a rodent consumed only its pulp or also its seed (25%, 50%, 75% or 100% seed mass consumption) and; (3) that a fruit/seed was cached under *M. coquimbensis*, in rock cavities or in open interspaces. All rodents were released at the point of when trials ended and all experiments were performed in accordance with relevant guidelines and regulations. Capture and maintenance procedures for these experiments were approved by the Ethics and Bioethics Committee of Universidad de La Serena (Informe de Ética/Bioética DIULS N° 048/13).

#### Seedling emergence

Because seedling emergence is strongly dependent on rainfall^26^, we examined the effect of environmental conditions (i.e., the context) on seedling emergence by conducting sowing experiments in two localities (*ca*. 50 km apart) with different moisture conditions: Juan Soldado (29°39′53′′S, 71°17′58′′W) and Temblador (29°28′8′′S, 71°18′20′′W). Juan Soldado is located at the centre of *M. coquimbensis*’ distribution; from 2011 until 2016, this area received an average of 79 mm of annual rainfall. The experiment was conducted in a ravine perpendicular to the Pacific Ocean with slopes that constantly funnel moisture-laden ocean mist-clouds upslope, bringing humidity that adds to the precipitation fallen as rain. This locality represents wet conditions, as it is always moist and most *M. coquimbensis* plants fruit every year (*pers. obs*.). Conversely, Temblador is in the northern end of the distribution, and received an average of only 40 mm of annual rainfall between 2011 and 2016. The experiment was set up on a flat-hill crest exposed to prevalent onshore winds, where the openness of the landscape prevents moisture from building up; Temblador thus represents dry conditions.

We examined how seed size interacts with environmental conditions (i.e., wet and dry conditions) in determining the probability of seedling emergence in different caching habitats. To this end, we determined in each locality, the rate of seedling emergence in rock cavities and under conspecific shrubs by sowing a group of 30 seeds in each microhabitat replicate (N = 7 per microhabitat) in 2015. Each replicate consisted of 10 large (8.03–11.74 g), 10 medium (4.66–7.94 g) and 10 small (2–4.52 g) seeds that were superficially buried to mimic caching and were protected by a 25 × 20 × 10 wire cage (5 × 5 mm mesh size) to prevent rodents from removing seeds. Emergence was monitored once a month for six months. No seeds were sown in open interspaces because the probability of emergence in this habitat is nil^26^.

#### Emergence probability of partially predated seeds

To test the extent to which partial seed consumption affects seedling emergence, we simulated the effect of rodent consumption by experimentally removing portions of the seed’s cotyledonary reserves. Specifically, we selected 36 seeds (1.21–7.16 g) and randomly assigned each seed to one of four cutting treatments: (1) intact seeds (no cutting); (2) 25% seed mass loss; (3) 50% seed mass loss or; (4) 75% seed mass loss^62^. Intact seeds and cotyledon fragments were sown in individual plastic pots in greenhouse conditions and watered regularly. We checked for emerged seedlings once a week for 6 months.

## Simulation Model

**Figure 4:**
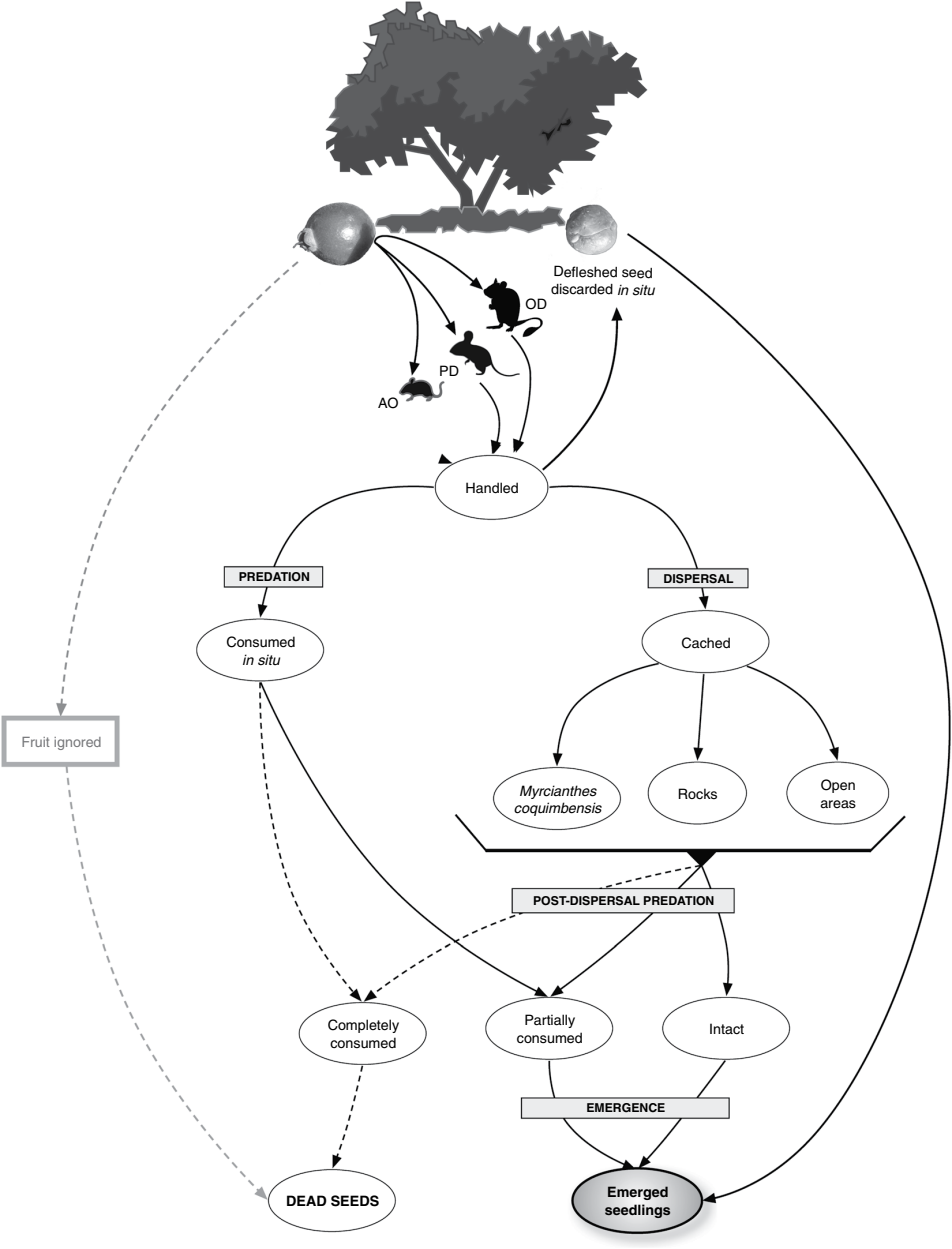
Flow diagram depicting the possible fates of *M. coquimbensis* fruits handled by *Abrothrix olivaceus*, *Phyllotis darwini* or *Octodon degus*. Dashed lines indicate transitions that lead to seed death. Fate of seeds from fruits ignored by rodents (denoted by gray lines) were not considered in this study since pulp removal is necessary for seedling emergence. Shrub and rodent vectors were purchased from vecteezy.com.

We used a stochastic model adapted from the model used by Calviño-Cancela and Martín-Herrero^63^. It consists of a series of recruitment stages connected by transition probabilities (Fig. 4). Specifically, the model incorporates the probabilities that seeds of different size categories are discarded, cached or consumed by each rodent species (see *Rodent Behaviour* subsection above). For selected seeds, we incorporated the probability of either being consumed at the site of encounter (i.e., beneath the mother plant) or dispersed (cached) to different microhabitats (rock cavities or open interspaces), as well as the probability of cached seeds of being subsequently ignored or consumed at the caching sites. For consumed seeds, we incorporated the probabilities of 25%, 50%, 75% or 100% seed mass consumption. For seeds remaining alive (i.e., partially consumed or intact), we incorporated the probability of seedling emergence in the different microhabitats (see the Seedling emergence subsection above), weighted by the effect of partial predation on seedling emergence (see Emergence probability of partially predated seeds subsection above). For each transition, we had a set of probabilities equal to the number of replicates in each of the experiments. Simulation outputs for each iteration (N = 500 iterations) were calculated as the product of randomly selected transition probabilities at each stage (e.g., the probability of being handled by a rodent × the probability of being cached in rock cavities × the probability of being subsequently consumed with a 25% loss of seed mass × the probability of seedling emergence in rock cavities, weighted by the effect of 25% seed mass loss in emergence). For the random selection of the transition probabilities in each iteration, we used a multistage resampling strategy based on bootstrap^64^ that does not require any assumptions about the statistical distribution of the transition probabilities. In contrast to deterministic models, stochastic simulations consider the stochastic nature of the process, better reflecting the nature and complexity of the recruitment process. The model allowed us to track the fate of seeds along the stages of recruitment and to examine the overall effect of rodents on the recruitment success of this anachronic plant species through their role as predators and/or dispersers.

### Statistical analyses

The effect of environmental conditions (wet vs. dry), seed size and handling (i.e., whether the seed was discarded, consumed or dispersed and the identity of the rodent species handling the seed) on recruitment success of *M. coquimbensis* was analysed using a Generalized Linear Model with a binomial error distribution (link function = logit). Averages are shown with standard errors all throughout the text.

## Data Availability

Data is available at https://doi.org/10.5061/dryad.c866t1g3q
